# Fragile mitophagy in long COVID: a proposed recovery-failure endotype for post-exertional malaise

**DOI:** 10.3389/fmed.2026.1905758

**Published:** 2026-08-12

**Authors:** Robert Groysman

**Affiliations:** Independent Researcher, Covid Institute, Plano, TX, United States

**Keywords:** endotype, fragile mitophagy, long COVID, lysosome, mitochondrial DNA, mitochondrial dysfunction, mitophagy, post-exertional malaise

## Abstract

Long COVID is frequently characterized by exertion intolerance, delayed symptom exacerbation, treatment sensitivity, and prolonged recovery. Post-exertional malaise (PEM) is often interpreted as a manifestation of low energy availability, autonomic dysfunction, immune activation, endothelial disturbance, or deconditioning. This paper proposes an additional recovery-failure mechanism for a PEM-dominant subgroup: fragile mitophagy, defined as a mismatch in which mitochondrial injury and mitophagy engagement occur but lysosomal completion of mitochondrial degradation is inadequate. Incomplete clearance could permit mitochondrial debris and danger signaling to persist and amplify oxidative, innate immune, endothelial, and neuroimmune responses after physiologic stress. The model predicts delayed crashes, progressive lowering of baseline with repeated exertion, and poor tolerance of interventions that increase mitochondrial turnover when lysosomal capacity is insufficient. Patient-derived Long COVID studies demonstrate mitochondrial, metabolic, and structural abnormalities but do not yet establish defective dynamic mitophagy flux or lysosomal completion. Hydroxychloroquine and chloroquine are used only as pharmacologic analogies showing that late autophagic flux can be impaired by disrupted lysosomal handling. Trehalose, genistein, curcumin, and the curcumin analog C1 are presented as experimental mechanistic probes because of reported effects on TFEB or autophagy-lysosome biology, not as established Long COVID treatments. The central prediction is that PEM severity will correlate more closely with impaired lysosomal completion, abnormal mitophagy flux, mitochondrial debris, and danger signaling than with baseline adenosine triphosphate (ATP) deficiency alone.

## Introduction

Long COVID involves heterogeneous mechanisms, including immune dysregulation, autonomic dysfunction, endothelial injury, viral or antigen persistence in selected contexts, metabolic disturbance, mitochondrial abnormalities, mast-cell activation, and neurovascular dysfunction. Reviews and patient-derived studies support mitochondrial involvement in fatigue and exercise intolerance, but findings differ by tissue, cell type, disease stage, and assay method (1–5). A 2024 skeletal muscle study found lower oxidative phosphorylation capacity and worsening muscle abnormalities after PEM induction, supporting the interpretation that PEM reflects measurable post-exertional biology rather than deconditioning alone (3).

The novel contribution of this paper is the proposal that PEM may reflect failure of mitochondrial recovery and lysosomal completion, rather than mitochondrial energy deficiency alone.

These observations raise an important question: why do some patients deteriorate after exertion rather than adapt to it? In healthy individuals, exertional injury is buffered by antioxidant defenses, mitochondrial dynamics, selective quality-control pathways, lysosomal degradation, and mitochondrial biogenesis. The fragile mitophagy hypothesis proposes that delayed crashes occur when exertion increases mitochondrial injury and turnover requirements beyond the capacity of the downstream degradative system (1–3, 6, 7).

SARS-CoV-2 biology provides mechanistic plausibility for disruption of autophagy-lysosome pathways. ORF3a, ORF7a, NSP6, and other viral proteins have been reported to alter autophagosome-lysosome fusion or related trafficking in experimental systems (8–10). Most of this evidence comes from cell culture, acute infection, or other preclinical models. It does not establish that viral-protein-mediated lysosomal dysfunction persists in Long COVID or causes PEM. Persistent viral or antigenic effects are therefore treated as one possible contributor rather than a necessary component of the model.

In Long COVID, recovery capacity may be reduced by converging stressors, including immune activation, oxidative stress, dysautonomia, endothelial or microvascular dysfunction, sleep disruption, metabolic strain, mast-cell or neuroimmune activation, and viral or post-viral cellular disturbance. These mechanisms may increase mitochondrial injury, reduce lysosomal clearance capacity, or both, placing fragile mitophagy within a broader interacting network rather than in competition with other explanations for PEM (1–3, 11).

Several nonexclusive mechanisms could impair lysosomal completion, including post-infectious inflammation, oxidative stress, altered lysosomal acidification or endolysosomal pH, impaired protease activity, disrupted trafficking, and persistent cellular stress (8–10, 12, 13). These are biologically plausible routes rather than established or universal abnormalities in Long COVID. The proposed mismatch requires only that mitochondrial injury load exceed lysosomal clearance capacity in a susceptible subgroup.

Myalgic encephalomyelitis/chronic fatigue syndrome (ME/CFS) is an important comparator because both conditions can include delayed PEM, prolonged recovery, exertional intolerance, and mitochondrial or metabolic abnormalities. In a small exploratory muscle-biopsy study, both chronic fatigue syndrome and post-COVID groups had fatigue and mitochondrial abnormalities; the post-COVID group showed reduced complex I activity relative to healthy controls, whereas the chronic fatigue syndrome group had more pronounced cristae and structural abnormalities than the post-COVID group (14). These findings suggest overlap without establishing mechanistic identity, and similar clinical phenotypes do not prove the same mitochondrial-clearance defect (15). Comparative studies should determine whether impaired mitophagy completion is shared across these conditions, restricted to particular subgroups, or specific to selected post-infectious states.

## Hypothesis

This paper extends a mechanism-anchored network framework for Long COVID by proposing that fragile mitophagy may define one experimentally testable convergence endotype within that network (11). It is not a single-cause explanation for Long COVID or PEM. Dysautonomia, endothelial or microvascular dysfunction, immune activation, oxidative stress, mast-cell reactivity, metabolic disturbance, and other stressors may converge by increasing mitochondrial injury or reducing cellular clearance capacity. Incomplete mitochondrial recovery may then amplify inflammatory, endothelial, neuroimmune, and autonomic abnormalities.

Mitochondrial quality control extends beyond PINK1/Parkin-mediated mitophagy. It also depends on coordinated fission and fusion through proteins such as DRP1, MFN1/2, and OPA1; receptor-mediated mitophagy involving BNIP3/NIX, FUNDC1, and PHB2; selective removal of damaged cargo through mitochondrial-derived vesicles; and replacement through PGC-1α- and TFAM-regulated biogenesis (6, 7, 16–19). Failure at any step could increase damaged mitochondrial burden or place additional demand on lysosomal degradation.

Mitophagy itself is a multi-step process (6, 7). Damaged mitochondria must be recognized, segregated, engulfed, delivered to lysosomes, degraded, and recycled. The central hypothesis is that initiation may remain active or become upregulated while late lysosomal degradation is delayed or insufficient. When initiation exceeds completion, mitochondrial fragments, mitochondrial DNA, oxidized lipids, cardiolipin, and damaged proteins may persist as danger signals.

The key clinical prediction is that patients with this endotype will show impaired recovery after mitochondrial stress. The expected phenotype is PEM-dominant, with delayed and prolonged crashes, worsening after repeated exertion, poor tolerance of aggressive mitochondrial stimulation, and improved tolerance only when injury load and downstream clearance are better matched (3).

## Fragile mitophagy as a proposed recovery-failure endotype

Fragile mitophagy is proposed as a functional mechanistic endotype rather than a currently validated clinical diagnosis. The observable phenotype is delayed and prolonged PEM; the proposed endotype is a mismatch between mitochondrial injury, mitophagy engagement, and lysosomal completion. Its prevalence, clinical boundaries, tissue distribution, and temporal relationship to PEM are unknown. Existing patient-derived Long COVID studies support mitochondrial abnormality in selected cohorts but do not directly demonstrate this proposed completion defect (1–5, 20).

Reduced ATP production may explain fatigue during activity, but it does not fully explain why symptoms often worsen 24–72 h later or why recovery may take days. The delayed component of PEM is hypothesized to reflect a secondary cleanup and inflammatory amplification phase. Incompletely cleared mitochondrial DNA, cardiolipin, oxidized lipids, damaged proteins, and mitochondrial fragments may activate innate immune pathways, including cGAS-STING and the NLRP3 inflammasome (21–23).

Mitochondrial DNA can reach the cytosol through several experimentally described routes, including BAX/BAK macropore formation with inner-membrane herniation and VDAC oligomerization (24, 25). These routes have not been demonstrated as drivers of PEM in Long COVID; they are included only to specify plausible links between mitochondrial injury and innate immune sensing.

The term recovery reserve is used here to describe the practical physiologic capacity to tolerate a stressor, complete mitochondrial cleanup, and return to baseline. A patient with preserved recovery reserve may respond to controlled stress by activating repair pathways. A patient with poor recovery reserve may respond to the same stimulus with prolonged worsening. In this paper, PEM is proposed as the clinical readout of poor mitochondrial recovery reserve.

## How fragile mitophagy could produce PEM 24–72 h later

PEM is the most recognizable clinical expression of the proposed endotype. It is a delayed and prolonged worsening after physical, cognitive, orthostatic, emotional, thermal, or infectious stress rather than ordinary fatigue after activity. Symptoms may include severe fatigue, flu-like malaise, cognitive dysfunction, sleep disruption, pain, heaviness, tachycardia, dizziness, sore throat, headache, sensory sensitivity, and a lowered threshold for subsequent crashes.

**Figure 1:**
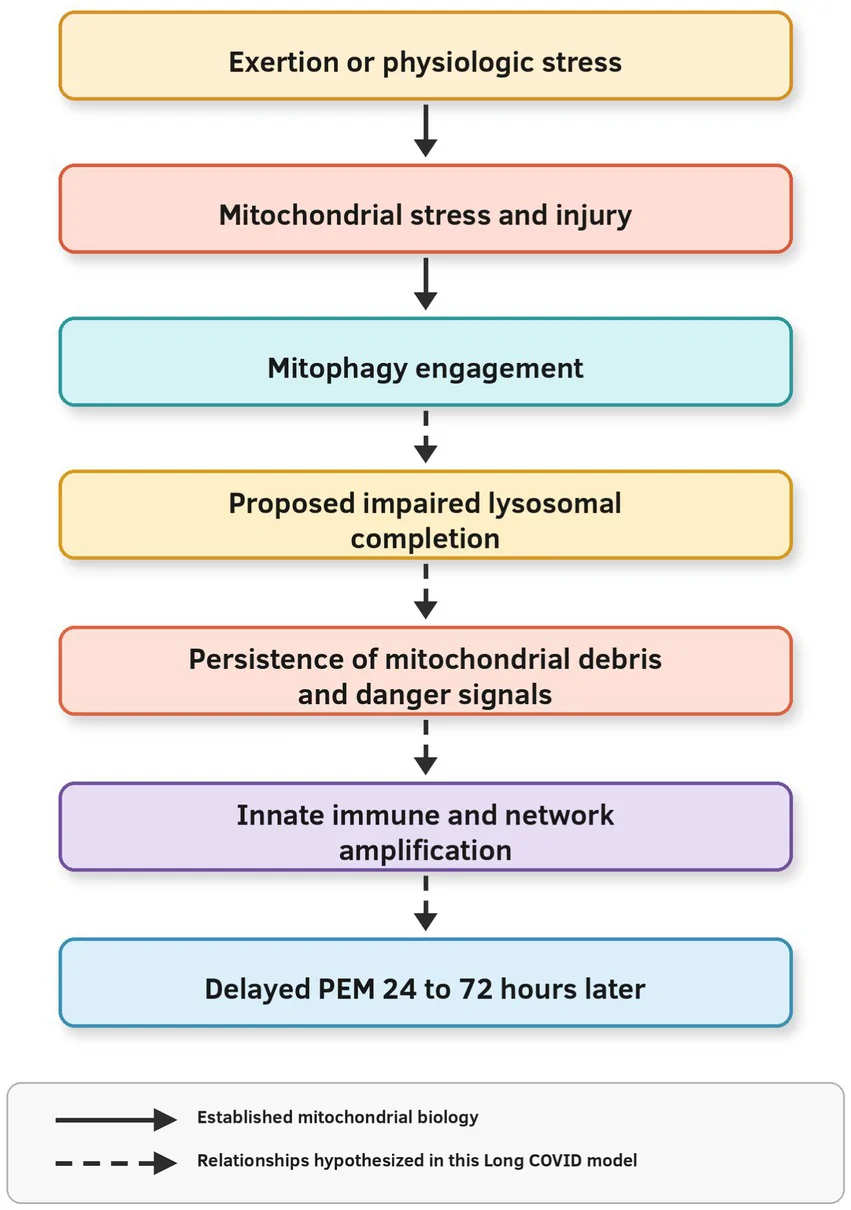
Proposed fragile mitophagy sequence linking exertion to delayed post-exertional malaise. The diagram is a simplified linear representation of one pathway within a broader interacting network. Solid arrows denote established relationships between physiologic stress, mitochondrial injury, and mitophagy engagement. Dashed arrows denote relationships hypothesized specifically in this Long COVID model, beginning with the proposed mismatch between mitophagy engagement and lysosomal completion. Impaired lysosomal completion in Long COVID is therefore a proposed mechanism rather than an established abnormality. The delay is hypothesized to reflect amplification kinetics across incomplete clearance, mitochondrial danger signaling, and downstream immune and neurovascular responses.

Figure 1 summarizes the proposed sequence. Exertion increases mitochondrial workload and injury. Mitophagy may be initiated, but lysosomal completion may be delayed, insufficient, or overloaded. Persistent mitochondrial debris and danger signals may then amplify innate immune, endothelial, neuroimmune, mast-cell, and autonomic responses (6, 7, 21–23).

In Figure 1, the transition from mitophagy initiation to impaired lysosomal completion is shown as hypothesized because defective lysosomal completion in Long COVID remains the central proposed mechanism of this model rather than an established finding.

PEM may appear 24–72 h after exertion because the proposed sequence requires time for injury to accumulate, clearance demand to exceed capacity, danger signaling to develop, and downstream systems to amplify the response. The delay is not presented as a single established biologic clock, but as a testable consequence of linked processes rather than immediate ATP depletion alone (3).

## Why repeated exertion may lower baseline

This framework may also explain why repeated exertion can progressively lower a patient’s baseline. If each exertional episode increases mitochondrial clearance demand, but clearance remains incomplete, then repeated activity may stack unresolved cellular stress. The patient may appear to have reduced conditioning, but the primary abnormality may be impaired recovery biology rather than insufficient training stimulus, consistent with findings that biological muscle abnormalities can worsen after PEM induction (3).

In this model, pacing is interpreted as a strategy to keep mitochondrial injury and turnover demand below the patient’s current recovery capacity, rather than as avoidance of activity alone. If lysosomal completion improves, the threshold for activity tolerance should rise. If mitochondrial injury repeatedly exceeds clearance capacity, the threshold for PEM should fall.

## Clinical screening framework

Clinical screening should begin with the observable PEM-dominant phenotype and then test whether mitochondrial injury, mitophagy flux, and lysosomal function support the proposed endotype. The purpose is to identify candidates for research evaluation, not to make a clinical diagnosis from symptoms alone (3, 4, 6, 7). Table 1 summarizes the proposed framework.

**Table 1:** Proposed clinical screening and research testing framework for fragile mitophagy.

Screening domain	Examples	Potential clinical relevance
Clinical screening features	Delayed PEM 24–72 h after exertion; prolonged recovery; worsening with repeated exertion; intolerance to aggressive exercise, fasting, heat stress, or strong mitophagy-promoting interventions	Identifies patients who may have impaired mitochondrial recovery capacity rather than simple fatigue or deconditioning
Supportive laboratory markers	Lactate, pyruvate, creatine kinase, lactate dehydrogenase, acylcarnitines, plasma amino acids, urine organic acids, glutathione status, F2-isoprostanes, 8-hydroxy-2′-deoxyguanosine	May provide nonspecific evidence of mitochondrial, metabolic, redox, or oxidative stress, but is not diagnostic; no validated cutoff confirms fragile mitophagy.
Research assays	Circulating or cellular mitochondrial DNA measures, mitochondrial ROS, mitochondrial membrane potential, Seahorse extracellular flux analysis, dynamic LC3-II/p62 flux, TOM20-LAMP1 colocalization, mito-Keima or tandem mitochondrial reporters, lysosomal pH assays, cathepsin activity, DQ-BSA proteolysis, TFEB localization	Evaluates mitochondrial injury, dynamic autophagy/mitophagy flux, and lysosomal function; requires validation and standardization and is not a routine clinical test.

ROS, reactive oxygen species; LC3-II, lipidated microtubule-associated protein 1 light chain 3; p62, sequestosome 1; TOM20, translocase of the outer mitochondrial membrane 20; LAMP1, lysosome-associated membrane protein 1; DQ-BSA, dye-quenched bovine serum albumin; TFEB, transcription factor EB.

A practical research screen should document whether exertion produces a delayed crash, whether recovery requires days rather than hours, whether repeated activity lowers baseline, and whether strong exercise, prolonged fasting, heat stress, or interventions intended to increase mitochondrial turnover are followed by disproportionate worsening. These reactions are nonspecific and may arise through other mechanisms; they should be used only to enrich prospective cohorts, not to diagnose the endotype or select treatment (3, 26–29).

## Laboratory and research testing

No currently available routine clinical laboratory test confirms fragile mitophagy, impaired mitophagy completion, or lysosomal failure in Long COVID. Paired lactate and pyruvate, creatine kinase, lactate dehydrogenase, acylcarnitines, plasma amino acids, urine organic acids, glutathione status, and oxidative-stress markers may identify metabolic or redox abnormalities, but none directly measures mitophagy completion or has sufficient specificity to establish the proposed endotype (4).

Research-level testing should evaluate the full clearance pathway. Candidate assays include mitochondrial reactive oxygen species and membrane potential, Seahorse extracellular flux analysis, TOM20-LAMP1 colocalization, lysosomal pH, cathepsin B/D/L activity, DQ-BSA proteolysis, and TFEB nuclear localization. Dynamic flux assessment is essential: isolated LC3-II, p62, LAMP1/2, LysoTracker, TFEB, or colocalization measurements cannot distinguish increased pathway engagement from impaired downstream degradation. LC3-II/p62 testing with and without lysosomal blockade, mito-Keima or tandem fluorescent mitochondrial reporters, and degradation endpoints provide stronger evidence of mitophagy completion (4, 5, 30, 31).

Patient-derived evidence remains indirect and partly discordant. Szögi et al. reported mitochondrial structural abnormalities and higher ATG4B levels in Long COVID, but a static ATG4B measurement does not establish impaired mitophagy flux (4). Macnaughtan et al. identified altered mitochondrial bioenergetics in peripheral blood mononuclear cells without directly measuring lysosomal completion (5). Two post-COVID cohorts reported lower rather than higher relative circulating cell-free mitochondrial DNA (4, 20), arguing against treating an elevated plasma mitochondrial DNA signal as a necessary feature. The available patient-derived studies cited here have not yet demonstrated the proposed initiation-completion mismatch using dynamic mitophagy and lysosomal assays.

Direct patient-derived studies specifically evaluating autophagic or mitophagic flux and lysosomal completion in Long COVID remain sparse. The available literature therefore does not yet provide a sufficiently developed body of positive and negative studies to establish a consistent disease-specific pattern.

Translation will require prespecified specimen collection and processing, exertional-challenge timing, cell-composition controls, medication documentation, assay thresholds, and reproducibility across laboratories. Validation should address tissue specificity, sensitivity, specificity, within-person variability, clinically meaningful change, feasibility, cost, and comparison with non-PEM Long COVID, ME/CFS, and other fatigue disorders. No validated diagnostic threshold or clinically meaningful change value currently exists for the proposed endotype.

Feasibility differs by specimen and method. Peripheral blood mononuclear-cell or monocyte assays permit repeated sampling but remain specialized translational surrogates and may not reflect skeletal muscle, vascular, neural, or tissue-resident immune abnormalities. Muscle biopsy may be more relevant to exertional pathology but is invasive, costly, and poorly suited to routine serial testing. A clinically useful biomarker would therefore require both mechanistic validity and a reproducible, accessible sampling strategy.

The most direct design would compare Long COVID patients with severe PEM, Long COVID patients without PEM, recovered post-COVID controls, and healthy controls. Sampling at baseline, immediately after a conservative submaximal challenge, and during the 24-, 48-, and 72-h recovery window could determine whether lysosomal or clearance abnormalities precede, follow, or reinforce PEM. Stratification should account for age, sex, acute illness severity, time since infection, reinfections, vaccination timing, comorbidities, medications, dysautonomia, ME/CFS criteria, and baseline activity. The model predicts greater post-exertional impairment of lysosomal function or mitochondrial clearance in the severe-PEM group even when baseline energy markers are similar (3–5).

## Hydroxychloroquine and chloroquine as pharmacologic analogies

Hydroxychloroquine should not be interpreted as a proposed cause of Long COVID or fragile mitophagy in this model. Long COVID is a multi-mechanism network disorder, not a simple consequence of lysosomal inhibition alone. Rather, hydroxychloroquine and chloroquine are used here as pharmacologic analogies demonstrating one isolated principle: late autophagic flux can be impaired when lysosomal handling is disrupted. Chloroquine and hydroxychloroquine are lysosomotropic agents used experimentally to interfere with autophagy. Mauthe et al. showed that chloroquine mainly inhibits autophagic flux by impairing autophagosome fusion with lysosomes (13). In the fragile mitophagy model, impaired lysosomal handling would be expected to contribute to PEM only when combined with increased mitochondrial injury load, oxidative stress, immune activation, autonomic stress, endothelial dysfunction, or other Long COVID network mechanisms.

This analogy illustrates the central principle of fragile mitophagy: mitochondrial cleanup is only therapeutic when the disposal system is functional. If damaged mitochondrial cargo is delivered toward lysosomal degradation but cannot be efficiently degraded, the cell may accumulate partially processed cargo rather than complete recovery. The same logic may apply to Long COVID patients with impaired lysosomal completion. The problem is not mitophagy itself. The problem is mismatch between initiation and completion (6, 7, 13).

## Treatment implications and therapeutic sequencing

The hypothesis predicts that candidate interventions affecting mitochondrial quality control may have different effects depending on lysosomal competence. In a patient with intact lysosomal function, increasing mitophagy may improve mitochondrial quality. In a patient with incomplete lysosomal degradation, the same intervention is predicted to increase mitochondrial turnover demand without ensuring clearance. This could theoretically worsen PEM, oxidative stress, and inflammatory signaling (26–29). Table 2 summarizes candidate intervention categories and their predicted roles within the fragile mitophagy model.

**Table 2:** Candidate intervention categories and predicted roles in the fragile mitophagy model.

Intervention category	Examples	Evidence basis	Predicted role in fragile mitophagy
Mitophagy/autophagy-promoting interventions	Urolithin A; spermidine; fasting-related autophagy; berberine	Urolithin A has been reported as a mitophagy activator in humans; spermidine and fasting promote autophagy; berberine has been reported to induce AMPK-linked mitophagy (26–29).	Predicted to improve mitochondrial quality control when lysosomal completion is intact, but to increase turnover demand without adequate clearance when degradative capacity is insufficient.
Mitochondrial stabilizers and redox-supportive agents	Melatonin; astaxanthin	These agents are supported mainly as mitochondrial protective or antioxidant modulators rather than strong mitophagy inducers (32, 33).	Predicted to reduce oxidative injury and improve mitochondrial resilience before any experimental increase in mitochondrial turnover.
Lysosomal-supportive or TFEB-targeting interventions	Trehalose, genistein, curcumin, synthetic curcumin analog C1	Experimental studies demonstrate activation of TFEB signaling and/or enhancement of lysosomal biogenesis, lysosomal metabolism, or autophagy-lysosome function. These effects have not yet been shown to improve Long COVID or PEM (34–39).	Predicted to increase lysosomal capacity and mitophagy completion if the model is correct; this role remains untested in Long COVID PEM.

The examples are restricted to agents whose cited evidence directly supports the category in which they are placed. Mitochondrial-targeted strategies that primarily influence biogenesis, substrate use, or NAD metabolism should be evaluated separately from mitophagy induction because their timing, downstream clearance requirements, and tolerability may differ. None of these categories has been validated as treatment for fragile mitophagy or Long COVID PEM (26–29, 32–39).

This classification should not be read as proof that any agent treats Long COVID or fragile mitophagy. It is a hypothesis-generating framework. The central issue is not whether a compound is good or bad, but where it acts in the mitochondrial quality-control pathway. A compound that increases mitochondrial turnover may help if downstream lysosomal completion is intact. It may worsen symptoms if the disposal pathway is overloaded or impaired.

## Lysosomal repair as a therapeutic prediction

If fragile mitophagy results from incomplete mitophagy due to impaired lysosomal completion, then interventions that improve lysosomal biogenesis, lysosomal acidification, autophagic flux, or transcription factor EB (TFEB)-mediated lysosomal function would be predicted to increase lysosomal clearance capacity. This prediction is based on established lysosomal biology and provides a testable therapeutic hypothesis for the proposed fragile mitophagy endotype rather than an established treatment recommendation for Long COVID (34–39).

Trehalose, genistein, curcumin, and the synthetic curcumin analog C1 have all demonstrated effects on TFEB signaling, lysosomal biogenesis, lysosomal metabolism, or autophagy-lysosome function in experimental models. Trehalose activates TFEB and stimulates the autophagy-lysosome biogenesis program. Genistein activates TFEB, enhances lysosomal metabolism, and corrects lysosomal defects in experimental disease models. Curcumin and C1 activate the TFEB-lysosome pathway and enhance autophagy and lysosomal biogenesis *in vitro* and *in vivo* (34–39).

The hypothesis proposed here is not that these agents have been shown to treat Long COVID or post-exertional malaise. Rather, if impaired lysosomal completion proves to be a key mechanism underlying fragile mitophagy, agents with experimentally demonstrated lysosomal-supportive properties would be logical candidates for mechanistic testing. Their ability to restore mitophagy completion, reduce mitochondrial danger signaling, or improve PEM in Long COVID remains unknown and requires prospective investigation.

If the model is correct, these interventions would be expected to act primarily by improving the downstream degradative phase of mitochondrial quality control rather than simply increasing mitochondrial activity. Improved lysosomal completion could then allow damaged mitochondrial cargo to be efficiently degraded, potentially making later mitophagy-promoting interventions better tolerated (34–39).

This model also predicts that treatment sequencing may matter. In patients with fragile mitophagy, aggressive mitophagy induction is predicted to worsen symptoms if used before lysosomal capacity is restored. A hypothesis-generating sequencing model would be: first reduce oxidative stress and stabilize mitochondria, then support lysosomal repair and autophagic flux, and only later consider cautious mitophagy stimulation if PEM, recovery time, and biomarker profile improve (26–29, 32–39). No staged mitophagy-escalation protocol has been validated for Long COVID PEM; the sequence proposed here is a testable prediction of the model, not an established therapeutic protocol.

## Testing the hypothesis

A hypothesis paper should generate predictions that can be tested. The fragile mitophagy recovery-failure model makes several falsifiable predictions:

- PEM severity should correlate more strongly with impaired lysosomal completion and mitochondrial danger signaling than with baseline ATP deficiency alone (3, 4, 21, 22).
- Patients with severe PEM should show higher lysosomal pH, lower cathepsin activity, impaired DQ-BSA proteolysis, abnormal LC3-II/p62 flux, reduced mitochondrial-lysosomal clearance, or increased mitochondrial debris compared with Long COVID patients without PEM (4, 6, 7).
- After exertional challenge, severe PEM patients should show greater worsening of mitochondrial injury markers, oxidative stress, mitochondrial debris or danger signaling, or inflammatory activation than matched controls (3, 4, 20–22).
- Patients with the greatest lysosomal impairment should have the poorest tolerance to strong mitophagy-promoting interventions (26–29).
- Lysosomal-supportive interventions should improve lysosomal function markers before or alongside clinical improvement (34–39).
- If lysosomal repair improves mitophagy completion, circulating or cellular mitochondrial damage markers, oxidative stress, and inflammatory signaling should decline, and tolerance to exertion or mild mitophagy-promoting interventions should improve (4, 20–22, 34–39).
- If lysosomal function improves without any improvement in PEM, mitochondrial danger signaling, or treatment tolerance, the fragile mitophagy model would be weakened (3, 4, 21, 22).

## Research design and future directions

A pilot study could enroll four groups: Long COVID with severe PEM, Long COVID without PEM, recovered post-COVID controls, and healthy controls. Baseline and post-challenge testing should combine validated PEM and symptom instruments, orthostatic measures, objective activity monitoring, recovery time, prior treatment tolerance, and a focused panel of mitochondrial and lysosomal assays. Recruitment and analysis should prespecify demographic and clinical strata, including age, sex, acute illness severity, time since infection, reinfections, vaccination timing, relevant comorbidities, medications, dysautonomia, ME/CFS criteria, and baseline activity. Longitudinal sampling is needed to determine whether impaired clearance precedes PEM, follows repeated post-exertional injury, or participates in a self-reinforcing cycle.

A second phase could use a randomized, placebo-controlled mechanistic trial of a lysosomal-supportive candidate. Eligibility should require a defined PEM phenotype and evidence of impaired lysosomal or clearance biology at baseline. The primary endpoint should be a mechanistic measure such as lysosomal pH, proteolytic capacity, or dynamic mitophagy flux rather than symptom response alone. Secondary endpoints could include PEM frequency and duration, time to baseline, objective activity, mitochondrial danger signaling, and response to a standardized low-intensity challenge. Trials should prespecify worsening and stopping criteria and stratify participants by baseline lysosomal impairment.

Because exertional challenges and mitochondrial-quality-control interventions may worsen PEM in susceptible participants, prospective studies should use conservative submaximal thresholds, rescue criteria, adverse-event monitoring, prespecified stopping rules, and no escalation until tolerability is established.

Early trials should test mechanism before broad symptom efficacy. The aim would be to determine whether a defined PEM-dominant subgroup has a measurable clearance defect and whether changing that defect alters the predicted downstream biology. Failure to change lysosomal function, mitophagy completion, PEM, or treatment tolerance would weaken the model.

## Counter-hypotheses, limitations, and alternative explanations

Several mechanisms could account for PEM and mitochondrial findings in Long COVID. Dysautonomia may impair oxygen delivery and increase adrenergic metabolic strain; endothelial or microvascular dysfunction may limit perfusion or oxygen extraction; immune and mast-cell activation may amplify exertional symptoms; and hormonal, metabolic, sleep, medication-related, or deconditioning effects may reduce stress tolerance. These mechanisms may act upstream of mitochondrial injury, downstream of it, or in parallel, and their presence challenges any attempt to attribute PEM to one pathway (11).

Oxidative stress, endoplasmic-reticulum stress, the integrated stress response, lipid peroxidation, and ferroptosis-related injury provide additional alternatives or amplifiers. In ME/CFS, ER-stress-associated WASF3 overexpression has been linked experimentally to disrupted respiratory supercomplex assembly, illustrating a mitochondrial impairment mechanism that does not require failed lysosomal completion (40). Long COVID studies reporting ongoing oxidative stress or oxidized-lipid abnormalities support redox injury but likewise do not identify fragile mitophagy as the cause (41, 42).

Deconditioning may reduce exercise capacity and may coexist with Long COVID, but it does not adequately explain delayed symptom worsening, prolonged post-exertional crashes, flu-like or immune-like symptoms, or worsening after repeated low-level exertion. The fragile mitophagy model therefore does not deny deconditioning as a contributor in some patients; it argues that deconditioning alone is insufficient to explain the delayed recovery-failure pattern that defines PEM (3).

The model does not exclude these mechanisms. It proposes that incomplete mitochondrial clearance may be one downstream convergence point in a subset of patients and may interact bidirectionally with upstream autonomic, vascular, immune, and metabolic abnormalities. The linear sequence in Figure 1 is therefore a simplification of a network process, not a claim that one pathway explains the entire illness.

Circulating and cellular mitochondrial DNA findings in Long COVID are not consistently elevated and remain limited, assay-dependent, and unvalidated as diagnostic markers. Results may vary by compartment, timing, tissue source, disease stage, processing method, and normalization strategy. Recent studies have reported lower relative circulating cell-free mitochondrial DNA in selected Long COVID cohorts, but these findings do not establish impaired mitophagy and may not generalize across phenotypes (4, 20). Normal circulating levels would not exclude local, intermittent, intracellular, or tissue-restricted danger signaling, while abnormal levels would require confirmation with flux and lysosomal-function assays.

The prevalence, demographic distribution, and temporal direction of the proposed endotype are unknown. Cross-sectional abnormalities cannot determine whether impaired clearance causes PEM, develops after repeated PEM, or reflects a third process. Prospective longitudinal studies are required, and no current clinical assay should be used to label or treat patients as having fragile mitophagy outside a research context.

Methodologically, peripheral blood assays are accessible surrogates rather than substitutes for skeletal muscle, endothelium, neural tissue, or tissue-resident immune cells. Static LC3-II, p62, LAMP1/2, LysoTracker, TFEB, colocalization, or mitochondrial DNA measurements cannot prove failed completion because they may reflect adaptive stress, altered cell composition, medication effects, disease stage, or assay timing. Cross-sectional associations cannot establish temporal direction. The strongest evidence would come from standardized longitudinal flux-based designs showing that exertion precedes a reproducible clearance defect, that the defect tracks PEM severity and recovery time, and that improvement in lysosomal completion precedes or parallels clinical recovery (30, 31).

## Discussion

This hypothesis links delayed intolerance after physiologic stress with abnormal mitochondrial recovery biology. Fragile mitophagy is proposed as one possible convergence endotype within a larger Long COVID network, while delayed PEM and treatment intolerance are phenotype markers that may identify candidates for testing (1–3, 11).

The model moves beyond ATP deficiency alone by asking whether damaged mitochondria can be cleared and replaced after stress. Existing Long COVID studies support mitochondrial abnormalities, but they do not establish a uniform autophagy-lysosome defect. Static biomarkers, circulating mitochondrial DNA, and experimental viral-protein findings are insufficient on their own; dynamic, longitudinal, challenge-based studies are required. Observed mitochondrial dysfunction in Long COVID does not by itself establish impaired mitophagy completion; that inference requires dynamic flux-based and lysosomal-function evidence, which is not yet available in routine clinical practice or in most current patient-derived studies.

The principal limitation is the absence of direct evidence that impaired lysosomal completion causes PEM. The hypothesis should therefore be tested prospectively with prespecified clinical and biomarker outcomes and revised or rejected if the predicted flux, lysosomal, and recovery abnormalities are not observed.

Accordingly, the lysosomal-supportive agents discussed in this manuscript should be interpreted as mechanistic probes and research candidates, not as clinical recommendations. Their experimentally demonstrated effects on TFEB or autophagy-lysosome biology do not establish that they restore mitophagy completion, prevent PEM, or treat Long COVID. Their inclusion makes the hypothesis testable by identifying interventions predicted to alter lysosomal function if the model is correct.

## Conclusion

Fragile mitophagy is a testable recovery-failure endotype for a subset of Long COVID patients with delayed PEM, prolonged recovery, repeated-exertion worsening, and treatment intolerance. It proposes a mismatch between mitochondrial injury and the capacity to complete mitochondrial clearance, not a universal cause of Long COVID.

Support for the model would require convergent evidence that PEM tracks with impaired lysosomal function and mitophagy completion, that these abnormalities worsen after exertion, and that improving clearance changes both biomarkers and recovery tolerance. If those predictions fail, the model should be revised or rejected.

## Data Availability

The original contributions presented in the study are included in the article/supplementary material, further inquiries can be directed to the corresponding author.
